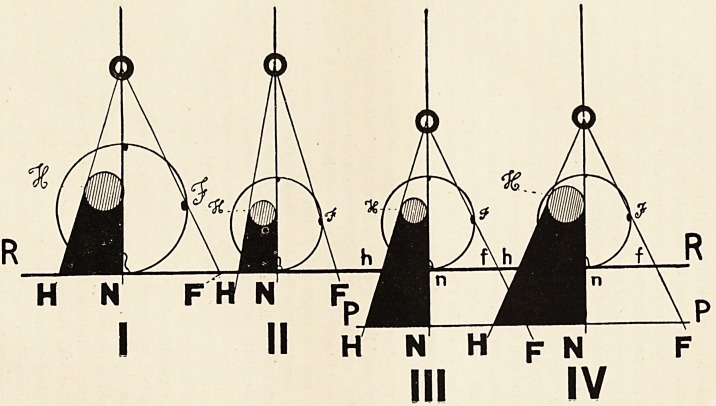# Proportional Representation and the Comparison of Radiographs

**Published:** 1907-12

**Authors:** William Cotton

**Affiliations:** Member of the Council of the Röntgen Society.


### PROPORTIONAL REPRESENTATION AND THE COMPARISON OF RADIOGRAPHS.

BY

WILLIAM COTTON, M.A., M.D., D.P.H.,

*Member of the Council of the Röntgen Society.*

NOT only does the radiograph of any part of the body need interpretation singly, but it may as regards the outlines depicted require to be compared with any other of the same part in others as one of a series of cases. In the attempts made up to now to adapt the radiographic art to clinical uses, one essential principle at least seems to have been almost entirely ignored, namely that (for reliable comparison of radiographs of corresponding parts) the distance of the focus tube from the parts to be represented should be proportioned to the size of these parts. In other words, corresponding parts of objects differing in size must be radiographed under equal angles if the resulting radiographs are to be at all comparable one with another.

The possibility at all of clinical radiography rests on the underlying assumption—till it is disproved—that in persons who are about the same age, and who are of the same sex, and of the same degree of fatness and leanness, but who differ in size, the corresponding regions of the body are in the geometrical sense *similar* to one another in their dimensions—that is, are enlarged or diminished models of each other—except in so far as their proportions may be altered by the effects of disease or of injury, or it may be by movement. This assumption is probably rarely quite true over the whole body, as where, for example, the man 5 feet 8 inches in height takes "tens" in boots, while his friend, who measures 5 feet 10 inches, gets along comfortably with "eight and a half." Yet so far as the feet are concerned, the bony skeletons of the larger feet are probably more likely to be *similar* to those of the smaller, each to each, than the containing pairs of boots, though these might very well be so too. One cuboid bone is to be regarded anatomically as *similar* to another. The assumption is probably approximately true in reference to the bones and bony distances of such segments of the axial and appendicular skeleton, and to such organs as the liver and heart, as are likely to come within the purview of the radiographer. According to this assumption, if we take two normal frozen bodies of the same age, sex and build, but differing in size, and if we make sections through them in various directions, we would expect on the surfaces of any pair of sections made in the same direction to be able to draw any number of triangles we please by a network of intersecting lines drawn between points corresponding anatomically, and we would expect to find by measurement that each triangle in the larger or smaller section would be *similar* respectively to the corresponding triangle in the smaller or larger section in every parallel pair of sections cut. Any dissimilarity observed would be the mark of abnormality.

But if this fundamental assumption be proved untrue, then there is an end at once to any exact use of radiography by the clinical surgeon or by the clinical physician. Nevertheless, for the purposes of the present argument, it is held to be *prima facie* true, and my present contention is that for proper comparison of radiographs *similar* parts in subjects of different size must be X-rayed under equal angles, that is at a distance of the focus tube from the parts proportional to the size of the parts.

In X-raying a part of the body for diagnosis, we virtually run a sheaf of sections simultaneously through the part of the body under examination, these sections being innumerable and radiating through a common Axis (the radiographic Axis), namely the particular ray that passes through the centre of the object we wish to take. At the same moment we *project* a sort of linear image of some of the contents of each intervening section upon the intersecting plane of delineation (plate or screen) by means of such of the rays emanating from the anticathodal point of emission (the radiographic Centre) as may survive the obstacles in their path. In radiographing for comparison the same region in a small man and a large one of the same age and build, if the Axis passes through corresponding anatomical points and the Centre is *similarly* situated to the parts, then the two resulting radiographs will be *similar* the one to the other on all parallel planes of delineation; and if the distance of the plane of delineation from the Centre of the focus tube in the one case is *equal* to the distance of the plane of delineation from the Centre of the focus tube in the other, then the resulting radiographs (barring morbid changes of size and shape) will not only be *similar* but *equal in every respect*, as might be proved by actual superposition. In geometrical parlance, the *projections* of *similar* solids on parallel planes by points *similarly* situated are *similar*, and may be *equal*.

In illustration of the foregoing remarks, I have tried in the accompanying diagram to show, as simply as possible, by means of four very conventional figures, what one might expect to find radiographically in the case of corresponding sections of three individuals, one of whom is larger than the other two, these two being equal in size externally. The larger circles represent sections of the thorax made transversely to the long Axis of the body, about the level of the fourth costal cartilage in front and the ninth dorsal vertebra behind, viewed from above. The smaller enclosed shaded circles represent the respective hearts. O is in each case the anticathodal Centre of emission, and O N is the radiographic Axis, drawn in each case through the right border of the sternum in front, and a point on the right side of the body of the ninth dorsal vertebra, proportionally distant from the mesial plane of the body. This Axis touches the right border of the heart, known to be *similarly* situated in I., II. and III. $\mathcal{H}$ is the extreme limit to the right

R
H N F
H N F
P
H N
H F N F
P
R
h
f h
f
n
n
I
II
III
IV

at the level chosen of the thoracic cage, namely an angular portion of the shaft of the fifth rib. The line R R represents the radiographic plane of delineation; the shorter transverse line P P parallel to the last, a plane of photographic enlargement of the radiographs in III. and IV., producing as it were (by appropriate use of the enlarging camera) the primary image in the X-ray plate to the same scale as the radiographs in I. and II.; O N (that is the distance of Centre from P P) in III. and IV. being made equal to O N in I. and II. (that is the distance of Centre from R R). The black represents in each case the shadow or *projection* H N and h n of the opaque hearts; and N F and n f the projection of *ℱ* and of all the intervening space between O N or O n and O F or O f. In all these cases the radiographic plate or screen is supposed to be as close to the posterior wall of the thorax as possible, *i.e.* in contact with the body according to the prevailing practice to secure greatest degree of clearness of image—and the Axis to be at right angles (what is called *normal*) to R R, so that when the radiographs were taken the distances between O and the remotest part of the body from O along the Axis and the distance of O from the plate coincided. It need not, however, have been so; theoretically for comparability of results the essential distance is the distance of O from the body at N (I. and II.) and n (III. and IV.); and the essential in regard to the plates is that they should be parallel to each other. Nevertheless it is of the highest practical convenience that the plates should be at right angles to the Axis at N, and as near N as possible. The distance of the parallel plates or screens from O is not essential as a matter of geometry, though it is of great importance for shortness of exposure that this distance should be as small as possible; but we do find that when the distance of the plate from O in one case is equal to what it is in another, then the two plates are on the same scale, and can be compared by superposition, provided always that the axial distance of the object from O in the one case is to that of the other *similarly* proportioned to the respective size of the objects.

In I. and IV. the hearts are equal in size; II. and III. are representations of the same individual.

In I. the axial distance of O from the remotest part of the body has been purposely made equal to twice the distance of 𝒮, from the tip of the spine of the eighth dorsal vertebra. It might, of course (subject to technical difficulties) have been made any other multiple of any other distance between bony landmarks; in practice (so long as we stick to the same multiple or sub-multiple of corresponding distances) no doubt in future it will be found convenient to take the distance between O and the nearest point of the body along the Axis, and make it proportional to the thickness of the body perpendicular to the plate. In II. the axial distance of O from the remote part of the body is the same length as in I.; but in III. and IV. the axial distance of O from the remote part of these bodies is a length the same multiple of the distance of 𝒮 from the eighth dorsal spine of these bodies, as O N is of the corresponding distance of 𝒮 there in IV. from the eighth dorsal spine of that body, namely 2.

These elements will be found tabulated in the annexed paradigm. The unit of measurement employed would amount to about a quarter of an inch in an actual radiograph of the chest.

Now it is evident that from the actual radiographs I., II., III. and IV., different radiographic experts would come to different conclusions with more or less reason on their side.

For instance, one radiographer would carefully measure H N and h n; he would make out the heart in III. to be somewhat and the hearts in I. and IV. greatly enlarged, taking no doubt the heart in II. as the normal, and acting on that curious principle in the morbid anatomy of the heart, whereby hearts always appear to be larger and never smaller than each other. But II. and III. are the same person; therefore the same object is at the same time larger and smaller, which is absurd. Another expert more philosophically would go by the ratios of H N or h n to N F or n f, N F and n f being the projections of ascertainable parts. He would probably make II. the standard, and regard the hearts of I. and III. as "equally enlarged" and of IV. as greatly so. On its being pointed out to him that II. and III. were from the same original, he would at once ask for the distance of O from the plate in each case ; and on learning that O N and O N in I. and II. were *equal*, and that O n and O n in III. and IV. were *equal* to each other, but shorter than O N in the other pair, he would put I. and II. apart from III. and IV. as belonging to two series not comparable with each other, and proceed to X-ray IV. anew, making the distance of O from n therein *equal* to what it was in I. and II.

How, then, are these discrepancies to be reconciled ? To avoid a task for which I feel personally unfitted of a rigid mathematical demonstration, I now venture under the form of an apologue to introduce a semi-mythical personage, whose methods of procedure are not unfamiliar to the readers of this *Journal*.

In a certain far country, lying towards the Great River, even the River Euphrates, there lived two learned hâkim or physicians. The one of them belonged to the sect of the millimetrists, the other relied on what he called common sense. Both had graduated and postgraduated with credit at a northern university of the Giaour, and had returned to practice the arts of medicine among their confiding kinsfolk at home. These two had met for the purpose of comparing some radiographs they had taken in the case of two or three of their patients. The patients complained of symptoms compatible either with indigestion or heart disease. The radiographs resembled the hypothetical cases of my text, and the two learned physicians came almost to blows over the very discordant indications thereon. At last, staggered by the discovery that II. and III. were from a patient whom they were both attending to at the same time, unknown to each other, they at length agreed to lay their differences before a certain wise man named Zadig, who had a great local reputation for setting down disputants and putting a full stop to people's troubles, and, in short, for seeing farther into a haystack than most. The last, having heard what they had to say with true oriental politeness, and having elicited from them some such particulars as these I have tabulated, retired into a small obscure chamber in the interior of his dwelling. There, having lighted a little taper of wax (which he kept near his bedside during the hours of darkness in case of being aroused by the robbers who infested that neighbourhood), he proceeded to throw on the white walls of his dark room the shadows of a sheep's heart and of some ribs of mutton (carefully denuded of flesh), which he happened to have by him. In doing so he watched attentively the various grazing contours made by his little cone of light upon the objects in his hand, as he passed his taper to and fro and back and forth. Having again emerged to the light of day, he sat awhile meditating, and drew on the sand with a twig some such diagrams like to those on page 329. Then slightly closing one eye, he thus addressed his expectant listeners in the sententious manner affected by eastern pundits :—

"Brothers, peace be with you. It is vain to fight about shadows. Life is short, art is long, and a straight line is the shortest. Truth lies on both sides. Clearly, my brethren, in II. the triangle O H N is not comparable with the triangles O H N and O h n in I., III. or IV., nor the triangle O N F there with O N F and O n f in the others, the two angles at O in II. being respectively unequal to the corresponding angles at O in the other three figures. Hence in II. the ratio of H N to N F cannot be compared to the ratio of H N to N F or of H n to n f in I., III. or IV., or in any other figures taken with the same proportional distance of O from the object, as in I., III. or IV. So, friends, you had best lay aside the radiograph II. ; it is but a single term of another series ; for I do now perceive, that the heart being unto X-rays a smooth and globular organ, unless it be radiographed under the same angle at O in all cases, it is the shadow of a different profile that will be cast in each case. Whereunto let the stereoscopists take heed.

"But in I. and III. the triangles O H N and O h n are both *similar* to each other, and likewise the triangles O N F and o n f ; so that H N : N F : : h n : n f. So I conclude that the hearts in these two men are not disproportionately enlarged as compared with the rest of their chests ; and I prognosticate that they will do well whenever you do cease to treat them. Praise be to Allah ! hearts are trumps, not spades. Further, I do reckon that the size of the heart in the one is to the size of the heart in the other directly as the size of the thorax in each.

"But in regard to the original of IV., I perceive he is in a very bad way, for I doubt not his heart is either much larger than it ought to be, or else it protrudeth out through the front of his bosom. If he live long enough, he will surely die. For though the triangles O H N and O h n in IV. are *dissimilar* to O H N and O h n in I. and III., yet verily the triangles O N F and O n f in I., III. and IV. are to each other *similar,* and the ratio of H N to N F and of h n to n f in IV. is much greater than the ratio of H N to N F and of h n to n f in I. and III. Nevertheless, I cannot tell you how much larger the heart of IV. is than the hearts of I. and III., unless you can assure me that the right border of the heart in IV. is *similarly* situated to that of the right border of the heart in I. and III."

So they prostrated themselves at the feet of the master, and departed marvelling, and disputing vehemently how they were to carry out the sage's behests, namely how in the case of IV. they might best make an angle h O n equal to the angle H O N or h O n in I. and III., in the new radiograph that they had it in their minds to make as Zadig commanded them.

Here the original MSS. becomes indecipherable, and the question remains, How did they manage to do it ?

To sum up, in radiographing the same part in different people, with the view of detecting the presence of dissimilarity by comparison of the members of the series one with another, I think it could be proved (*a*) that three things are geometrically necessary, and (*b*) that three things are radiographically expedient :—

(*a*) 1. The Axis must be directed through the same anatomical points ; one point is not sufficient.

2. The distance of the radiographic Centre from the part must be proportioned to the size of the part.

3. The planes of delineation must be parallel.

(*b*) 1. The plates or screens should be at right angles to the Axis, anatomically defined as above.

2. The distance of the plate or screen from the part should be as short as possible.

3. The resulting radiographs might with advantage be photographically enlarged (or diminished) to the same scale.

Although no doubt some apology is due from me to the mathematically inclined for a rather crude exposition of the doctrine of *similar* triangles, I trust this somewhat theoretical essay may lead to simpler methods of solving by radiographic delineation some perplexing clinical problems. Nor am I unmindful of the vexatious limitations imposed up to now on the X-ray worker by practical exigencies peculiar to the employment of the Röntgen rays in diagnosis. Yet any fine morning these technical difficulties may be overcome, and then a correct system of radiography will become of enormous importance. The risk of an incorrect system is not so much in missing excessive enlargement or displacement of parts, as in imagining abnormality to exist where it does not, or of failing to detect the lesser abnormalities. Till such a system be brought into uniform use, the medical witness would be wise to maintain in a court of law a *critical* attitude towards radiographs produced for his opinion, where he has not the means of checking the manner of their production in regard to the relative positions of Centre, Axis, Plate and Object.

### BIBLIOGRAPHY.

Euclid, *Elements of Geometry*, Books V. and VI.

## Figures and Tables

**Figure f1:**